# Amniotic Band Syndrome, Perinatal Hospice, and Palliative Care versus Active Management

**DOI:** 10.1155/2016/9756987

**Published:** 2016-11-29

**Authors:** Shadi Rezai, Justin Faye, Annika Chadee, Sri Gottimukkala, Ruchi Upadhyay, Carla Lara, Benamanahalli H. Rajegowda, Andrew D. Corwin, Rasila V. Lala, Jessica Vernon, Dilfuza Nuritdinova, Stephen Chasen, Cassandra E. Henderson

**Affiliations:** ^1^Department of Obstetrics and Gynecology, Lincoln Medical and Mental Health Center, 234 East 149th Street, Bronx, NY 10451, USA; ^2^St. George's University School of Medicine, True Blue, Grenada; ^3^Department of Obstetrics and Gynecology, Houston Methodist St. John Hospital, 18300 St. John Drive, Nassau Bay, TX 77058, USA; ^4^Department of Pediatrics, Lincoln Medical and Mental Health Center, 234 East 149th Street, Bronx, NY 10451, USA

## Abstract

*Introduction*. Amniotic band syndrome and sequence are a relatively rare condition in which congenital anomalies occur as a result of the adherence and entrapment of fetal parts with coarse fibrous bands of the amniotic membrane. A large percentage of reported cases have an atypical gestational history. The frequency of this obstetric complication is not affected by fetal gender, genetic abnormality, or prenatal infection.* Case*. A 21-year-old, G1P0 female parturient at 18 weeks and 5 days with a single intrauterine gestation during a routine ultrasound evaluation was noted to have amniotic band sequence. The pregnancy was subsequently complicated by preterm premature rupture of membranes with oligohydramnios, resulting in a surviving neonate scheduled for rehabilitative treatment.* Conclusion*. Amniotic band syndrome is an uncommon congenital anomaly resulting in multiple disfiguring and disabling manifestations. Several theories are proposed with most involving early rupture of the amnion and entanglement of fetal parts by amniotic bands. This syndrome can be manifested by development of multiple malformations, with the majority of the defects being limb abnormalities of a disorganized nature, as in the case we present. In the absence of a clear etiology of consequential congenital abnormalities, obstetric management guidelines should use shared decision models to focus on the quality of life for the offspring.

## 1. Introduction 

The clinical manifestations are primarily distal deformities, such as constriction of limbs and fingers, syndactyly, acrosyndactyly, phalangeal hypoplasia, pseudoainhum, and amputation of limbs and fingers [1, 2]. There is a predilection for the hand, in particular the central digits, whereas the frequency and severity of thumb involvement are minimal [3, 4]. The thumb is less vulnerable since it lies protected within the palm of the hand in utero, compared to the longer digits which are more exposed leading to amputations distal to the level of the proximal phalanx [3–6]. Multiple malformations such as clubfoot (30% of patients), leg length discrepancies (24%), other bone anomalies (12%), special craniofacial defects such as cleft lip and palate (8%), visceral and body wall defects, and anencephaly (5%) have been detected in 70% of infants with the disorder [7–11].

## 2. Case Presentation

A 21-year-old female, G1P0, presented to our clinic for an initial prenatal visit at 17 weeks and 3 days. Her medical history was significant for hemoglobinopathy AS and QuantiFERON positive without evidence of active tuberculosis. The initial sonogram at 18 weeks and 5 days gestation identified an anterior placenta, missing part of the left tibia and fibula, absence of the left foot, and floating bands strongly suggestive of amniotic bands (Figure 5), ending in a small extension of tissue of approximately 0.5–1.0 cm in length and 0.5 cm in width (Figures 2 and 4).

At 21 weeks in the emergency department, the fetus was in breech presentation with oligohydramnios and premature rupture of membranes (PPROM). After appropriate counseling about the outcome, the patient declined immediate pregnancy termination and elected antepartum admission for expectant management of PPROM. Intravenous hydration and antenatal corticosteroids were started at 27 weeks, due to nonreassuring fetal status. A 750-gram male infant was delivered via classical cesarean section with an Apgar score of 6 and 7 at 1 and 5 minutes, respectively. The immediate neonatal course was complicated by immaturity as manifested by respiratory distress syndrome, leading to intubation and admission to the neonatal intensive care unit.

**Figure 1:**
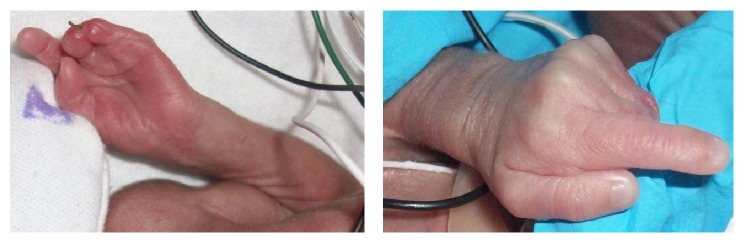
Left hand: newborn with visible bands tethering fingers together. Amniotic bands have resulted in syndactyly, amputation, adhesion, and band indentation. Thumb and index finger are intact; only proximal part of 3rd and 4th digit is present; 5th digit is completely missing.

**Figure 2 fig2:**
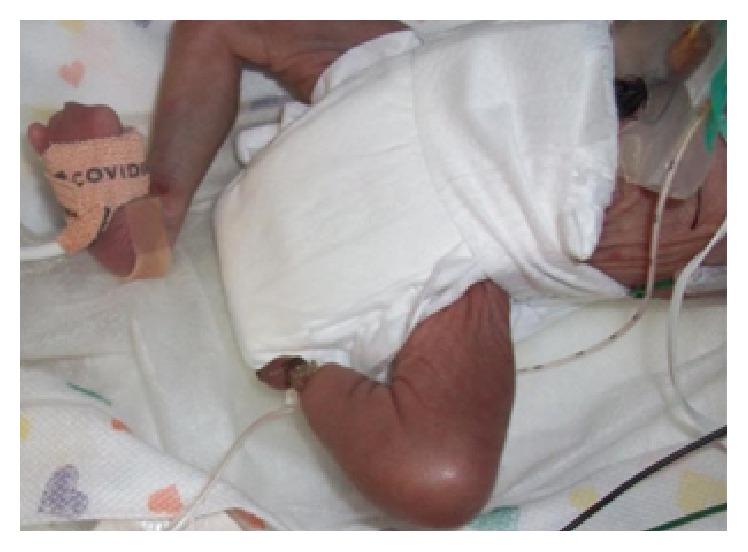
Left leg with amputation due to amniotic band. Right leg with only the 5th digit.

**Figure 3 fig3:**
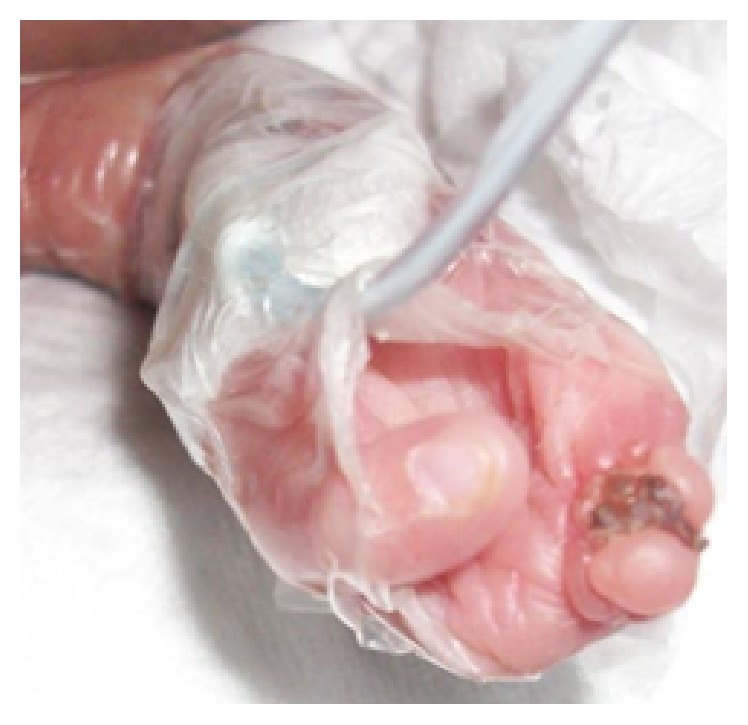
Right hand, only thumb present.

**Figure 4:**
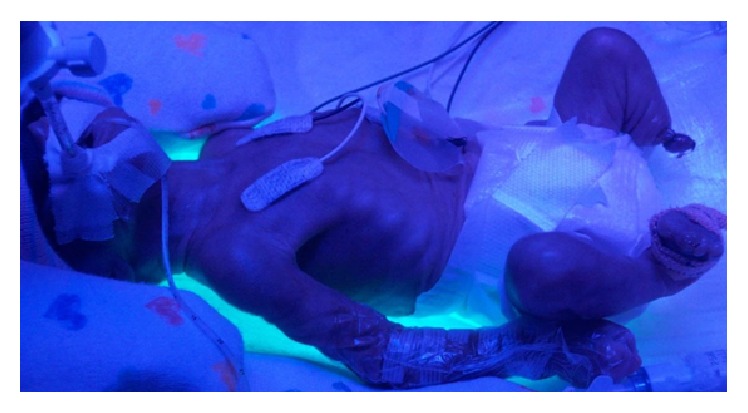
Under phototherapy, amputated left lower extremities are noted.

**Figure 5:**
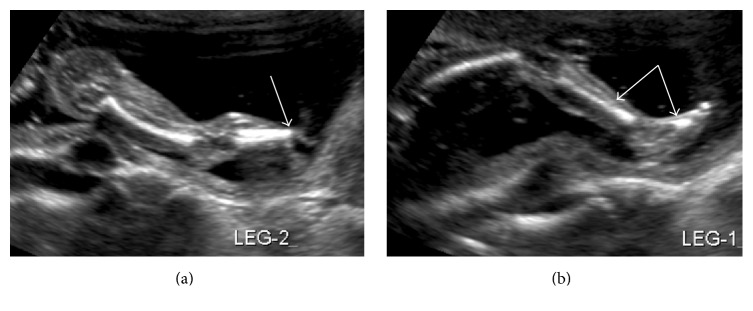
Official ultrasound on 1/13/2016: one lower extremity (left leg, leg 2 in (a)) is incomplete, the tibia fibula terminates abruptly and a foot is not seen. The other lower extremity (right leg, leg 1 in (b)) shows appropriate length for femur, tibia fibula, and a well formed foot. Noted during the examination are floating bands strongly suggestive of amniotic bands.

After thirty-six days of life, he is active and extubated, with equal breath sounds bilaterally, weighting 1120 grams and tolerating oral feeding while weaning from Total Parenteral Nutrition. Plans for future long-term care and physical therapy of his upper and lower limb abnormalities were initiated (Figures 1 [Fig fig2] [Fig fig3]–4).

## 3. Discussion 

There are two main theories as to the etiology of amniotic band syndrome. Originally, the intrinsic theory on inherent developmental defects was proposed by Streeter [12, 13]. The most likely explanation, which is supported by the majority of current authors, is the extrinsic theory proposed by Torpin [14–16]. It suggests adhesive bands around fetal limbs, due to slippage of the ruptured amnion from the chorion, leading to oligohydramnios and growth abnormalities [7, 15, 17, 18].

The diagnosis is based on ultrasound visualization of amniotic bands in an asymmetric distribution or deformities in a “random” nonembryonic distribution. These findings may be confirmed by fetal MRI, which is often ordered as a complimentary measure upon consideration of fetal surgery [13, 19, 20]. Amniotic bands are now being successfully released fetoscopically through minimally invasive surgery [19]. The intrauterine procedure is delicate and the decision to perform this surgery must be carefully weighed against the potential risks to the mother and the fetus versus continuing or terminating pregnancy [21–24].

Constricted bands are usually confined to the skin and soft tissues but at times can reach depths that cut off normal vascular and lymph supply, causing venous congestion, ischemia, chronic edema, clubfoot, and fractures of the afflicted areas [25–29]. When not diagnosed until postpartum, bands that interfere with drainage of the limb resulting in venous congestion or lymphedema can produce extreme pain, due to peripheral nerve compression requiring early surgical intervention repeated over several stages to improve long-term function [1, 28, 30–33].

## 4. Conclusion

The most widely held theory describes early rupture of the amnion and entanglement of fetal parts by amniotic bands, leading to congenital abnormalities most consistent with pathognomonic features of amniotic bands and limb/digit abnormalities or amputations. Due to the perinatal complications associated with intrauterine band lysis, multidisciplinary meeting consisting of family, obstetricians, neonatologist, psychologist, social worker, and other relevant entities should take place to discuss the options for continuation of pregnancy, perinatal hospice care, and pregnancy termination. Extrauterine surgical intervention should be performed within weeks to months, with a dedicated follow-up by family members of the newborn, pediatrics, and surgical teams to ensure functionality of the limb recently constricted, unless there is a vascular compromise, which would mandate an immediate surgical intervention. Due to the diverse presentation of amniotic band syndrome or ADAM complex, management should be tailored to the individual, taking into account the gestational age and birth defects at presentation.
